# Recognizing and managing anxiety disorders in primary health care in Turkey

**DOI:** 10.1186/1471-2296-11-30

**Published:** 2010-04-28

**Authors:** Mehtap Kartal, Ozlem Coskun, Nesrin Dilbaz

**Affiliations:** 1 Family Medicine Department of Dokuz Eylul University, 35340, Inciralti, Izmir, Turkey; 2 Medical Education Department of Gazi University, Ankara, Turkey; 3 The First Psychiatry Clinic, Ankara Numune Education and Research Hospital, Ankara, Turkey

## Abstract

**Background:**

Anxiety disorders are common and are frequently not diagnosed accurately in primary care. Our aim was to determine the knowledge gaps of general practitioners (GPs) in the diagnosis and treatment of anxiety disorders by using vignettes.

**Methods:**

A cross-sectional survey was completed with 255 primary care physicians (response rate 59.4%) in Manisa, a city in western Turkey. From the postal questionnaire, information on working experience, postgraduate education in psychiatry, the interests of the physicians in psychiatry were obtained. The physicians' diagnosis and treatment preferences for generalized anxiety disorder (GAD), social phobia (SP), and obsessive compulsive disorder (OCD) were determined through clinical vignettes prepared for data collection.

**Results:**

Two hundred and twenty-seven (89.0%) out of 255 GPs included the diagnosis of obsessive compulsive disorder in their differential diagnosis; however, the rates for social phobia and generalized anxiety disorder were 69.4% (n = 177) and 22.3% (n = 57), respectively. GPs with a post-graduate education on psychiatry diagnosed vignettes more accurately for OCD (p = 0.04). For all three cases, GPs mostly preferred a combination therapy including psychotherapy and psycho-pharmacotherapy. The referral rate to a psychiatrist was between 23.1 and 30.6%. The percentages of the prescription of selective serotonin reuptake inhibitors (SSRI) in accurate diagnosis were 59.3 for social phobia, 33.3 for GAD, and 55.5 for OCD.

**Conclusions:**

There is a gap of knowledge in GPs, which leads to poor recognition and management of anxiety disorders in primary care. Effective interventions including post-graduate education and updated guidelines on anxiety disorders should be planned and implemented with their assessments by vignettes.

## Background

Anxiety disorders are the most common disorders with a 12-month prevalence changing between 2.4 and 18.2% among the general population [1]. For the subgroups of anxiety disorders, the prevalence is 0.6-5.2% for social phobia (SP), 0.5-3.8% for generalized anxiety disorder (GAD), and 0.3-4.0% for obsessive compulsive disorder (OCD) [2-5].

Patients with anxiety disorders increasingly consult primary care units and this is reflected in the prevalence of anxiety disorders (14.6-19.0%), social phobia (2.6-12.3%), GAD (2.8-13.2%), and OCD (2.0%) in primary care [6-10].

In Turkey, although we do not have population-based prevalence studies for subgroups of mental disorders such as anxiety disorders, we know that more than 17% of the adult population has certain mental health problems. The National Burden of Disease Study results revealed that unipolar depressive disorder was ranked seven in the list of national disability-adjusted life year (DALY) calculations for the first 20 diseases [11,12]. It was estimated that only 13% of all the mentally ill patients could receive help for their problems [11].

Approximately 12-22% of the primary care patients present with symptoms of distress related to anxiety. General practitioners (GPs) identify about 50% of psychiatric disorders, and only one third of anxiety cases are correctly diagnosed [11,13]. The rate of accurate specific diagnoses for anxiety disorder is 35-65%, for pure GAD 34.4%, and for social phobia 24.0% [14,15]. These low rates may be due to patient-related reasons (such as unexpressed psychological problems, normalizing the symptoms, and the fear of stigmatization of mental illness), or physician-related reasons (such as unawareness of the diagnosis, inadequate recognition, competing demands in a limited time) [15-17]. Only 25% of the primary care patients with anxiety disorders received adequate medication, and less than 10% of them were counseled by a mental health professional [18].

In Turkey, undergraduate medical education starts after the university entrance examination following the secondary school, and lasts for 6 years. A basic doctor after graduation may practice in primary care as there is no necessity of residency or fellowship for implementation of practice. GPs work nearly in all health facilities including primary care centers, tuberculosis (TB) prevention dispensaries, maternal and child health centers, and the emergency services of state hospitals. There is no referral system from primary care, so patients can either be referred by their physicians in primary care or they can directly apply to all the hospitals, including those for mental health. Specialization in "Family Medicine" is still in its developing period with nearly 1,500 family medicine specialists working for health care.

The aim of this study was to examine the GPs' knowledge of anxiety disorders (social phobia, generalized anxiety disorder, and obsessive compulsive disorder) by clinical vignettes which are commonly used in the studies of depression and psychosis, and to our knowledge, which have not been done with anxiety disorders before.

## Methods

We conducted a postal survey for all GPs working in primary care institutions of the Ministry of Health (primary health care centers, maternal and child health centers, and dispensaries) in Manisa. Manisa is a city located in the Aegean region, with a population of 1,267,493 (Male 634,557, Female 632,936), 74.8% of which live in urban areas. In 2005, the health services in the city included 158 primary health care centers, 6 TB prevention dispensaries, 4 maternal and child health centers, 16 state hospitals, one children's hospital, one maternal hospital, and one mental health hospital. The mental health hospital is one of the oldest health facilities with a history beginning at 1539. It serves a total of 12 cities in the Aegean and West Mediterranean region with 400 beds.

As we do not have a specific questionnaire to assess GPs' knowledge in the diagnosis and management of anxiety disorders, we constructed a vignette-based instrument. Vignettes reflect a primary care presentation fulfilling Diagnostic and Statistical Manual of Mental Disorders, 4th edition (DSM-IV) diagnostic criteria for social phobia, generalized anxiety disorder, and obsessive compulsive disorder. The vignettes were assessed by three psychiatrists and finalized by a fourth one. The pilot study was conducted with ten psychiatrists and all the vignettes were diagnosed accurately. We also gathered additional information about the associated factors like working experience, postgraduate education on psychiatry and interest in psychiatry (Additional file 1).

The questionnaires and cover letters were posted to the sample in early January 2005 and were collected back in a month.

### Coding and analysis

GPs were asked to write a brief description of their treatment plan in an open-ended format for SP, GAD, and OCD. The management options were assessed in five categories: psychotherapeutic support and/or watchful waiting, referral to a psychiatrist, psychotherapy, psycho-pharmacotherapy, and combined therapy (psychotherapy and psycho-pharmacotherapy). GPs also had a paper for prescription in case they preferred to fill for the patient. However, as there was no information as to the duration of the medical treatment, we only assessed the accuracy of the prescriptions in three categories: accurate, acceptable, and unacceptable treatment according to the Diagnosis and Treatment Guideline for Primary Care, 2003[19]. The first-line treatment was named as accurate, the treatments other than the first-line were named as acceptable; however, the time period of the treatments could not be evaluated as they were not mentioned in the prescriptions. The agreement between two independent raters for the evaluation of prescriptions in line with the Diagnosis and Treatment Guideline for Primary Care, 2003, was found as 86.7% for SP, 78.5% for GAD, and 97.3% for OCD.

The data were installed and analyzed by the SPSS software, using descriptive statistics for frequencies, chi-square tests for the comparisons of independent proportions, with Fisher's exact test where appropriate. All the tests were two-tailed. The level of significance was set as p < 0.05. The prescriptions were reevaluated by two independent raters to report the agreement in terms of kappas.

## Results

The response rate was 59.4% (255 out of 429). Of the participants, 145 (56.9%) were male and 110 (43.1%) were female. The mean age was 36.6 ± 6.3 years; age was grouped as 26-35 (42.0%), 36-45(49.0%), and 46-55 (9.0%). The mean working experience was 12.3 ± 6.0 years, and 49.8% of the participants had a working experience 11-20 years, 40.4% were in group of 1-10 years. 42.7% of the participants mentioned that they had a postgraduate education on psychiatry, and 37.6% stated that they had an interest in psychiatry.

### Diagnosis

Two hundred and twenty-seven (89.0%) GPs included obsessive compulsive disorder in their differential diagnosis of OCD; however, the rate was 69.4% (n = 177) and 22.3% (n = 57) respectively for social phobia and generalized anxiety disorder. Other common diagnoses for OCD were psychosis (n = 5, 2.0%), paranoid disorder (n = 4, 1.6%), anxiety (n = 2, 0,8%); for SP they were antisocial personality disorder (n = 15, 6.0%), anxiety (n = 14, 5.6%), panic attack (n = 11, 4.4%); and for GAD they were panic disorder (n = 93, 37.8%), depression (n = 40, 16.3%) and post traumatic stress disorder (n = 21, 8.5%). Of all the GPs, 3.9% (n = 10) diagnosed all vignettes inaccurately, 25.9% (n = 66) diagnosed one vignette accurately, 55.7% (n = 142) diagnosed two vignettes accurately and only 14.5% (n = 37) diagnosed all the vignettes accurately.

There were no significant differences between the gender, age and interest in psychiatry of the GPs and the accurate diagnosis of anxiety disorders (p > 0.05). The GPs with a post-graduate education (97.1%) on psychiatry accurately diagnosed the OCD vignettes more frequently than those with no post-graduate education (90.0%) on psychiatry (p = 0.04).

### Treatment and Referrals

**Figure 1:**
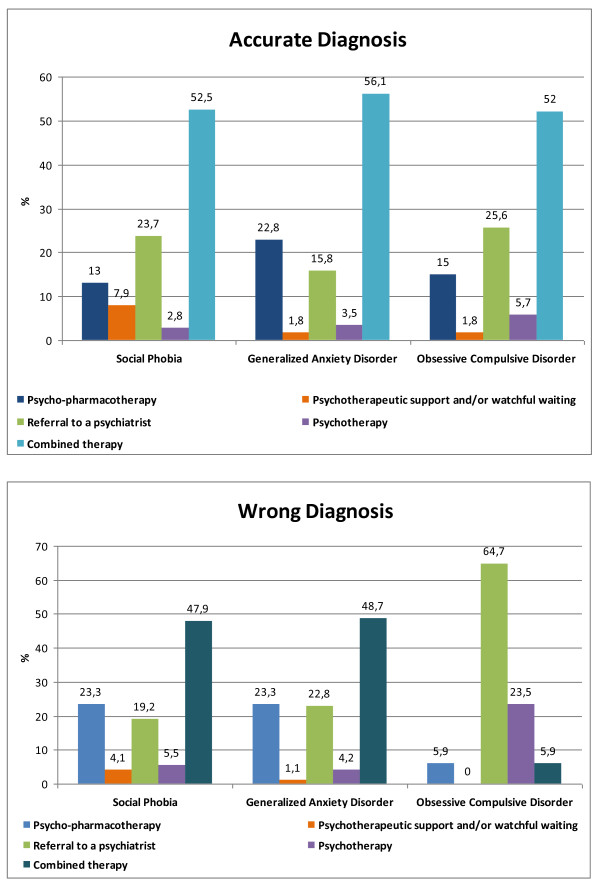
**GPs' treatment choices for the vignettes according to the accuracy of the diagnosis**.

The treatment preferences of the GPs for the vignettes, according to the accuracy of their diagnosis, were shown in Figure 1. For all three vignettes diagnosed accurately, the GPs mostly preferred a combination therapy including psychotherapy and psycho-pharmacotherapy. There was no significant relationship between the gender and the age of the GPs and the treatment options (p > 0.05). The referral rates of the male GPs were 29.0% and 36.6% for GAD (p = 0.007) and OCD (p = 0.02), respectively, which were higher than those of the female GPs (14.5% and 22.7%). The GPs with a post-graduate education and interest in psychiatry prefer referral for OCD (20.2%; 17.7%), which were lower than those with no education (38.4%; p = 0.002) and interest (38.4%; p = 0.0001). For GAD, this is also true with percentages of 8.3% and 33.6% for post-graduate education (p = 0.0001) and 15.6% and 27.0% for interest in psychiatry (p = 0.045), respectively.

The GPs with accurate diagnoses (25.6%) were less likely to refer the patient compared to those GPs with inaccurate diagnoses (64.7%) for OCD vignette (p = 0.001), who preferred the referral more.

### Prescribing

In SP, GAD and OCD vignettes, the GPs preferred not to prescribe any drugs with a rate of 33.3%, 27.8%, and 37.6%, respectively, and they preferred psychotherapeutic support and/or watchful waiting or referral to a psychiatrist or psychotherapy (Table 1).

**Table 1:** The treatment preferences of GPs for anxiety disorders

	Social Phobia % (n)	Generalized Anxiety Disorder % (n)	Obsessive Compulsive Disorder % (n)
No prescription	33.3 (85)	27.8 (71)	37.6 (96)

Psychotherapeutic support and/or watchful waiting	18.8 (16)	2.8 (2)	2.1 (2)
Referral to a psychiatrist	69.4 (59)	81.7 (58)	81.3(78)
Psychotherapy	11.8 (10)	15.5 (11)	16.7 (16)

Prescription	66.7 (170)	72.2 (184)	62.4 (159)

Prescribed drugs*			

	% (n^a^)	% (n^b^)	% (n^c^)
SSRI	43.5 (97)	48.8 (140)	62.5 (120)
Benzodiazepines	8.5 (19)	23.3 (67)	6.3 (12)
TCA	7.6 (17)	6.3 (18)	25.5 (49)
MAO inhibitors	21.5 (48)	1.0 (3)	0.5 (1)
B blocker	6.7 (15)	4.9 (14)	0.0 (0)
SNRI	2.2 (5)	3.1 (9)	2.1 (4)
Antipsychotic	0.0 (0)	2.4 (7)	0.5 (1)
Others	9.9 (22)	10.1 (29)	2.6 (5)

Number of drugs prescribed: ^a ^n = 223. ^b ^n = 287.^c ^n = 192.

* More than one drug is prescribed

**Figure 2:**
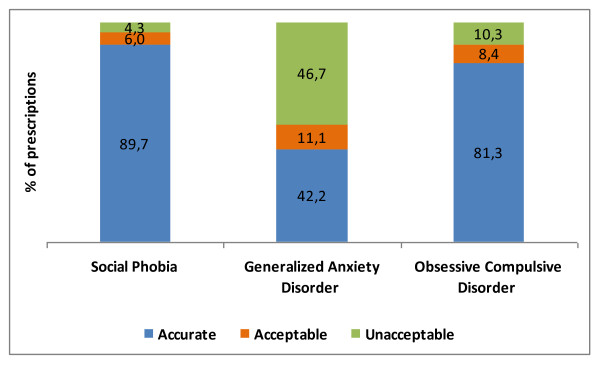
**The evaluation of the prescriptions of GPs who diagnosed vignettes accurately**.

For all anxiety cases, selective serotonin reuptake inhibitors (SSRI) were the first-line drugs preferred by the GPs. The accuracy of prescriptions were evaluated as accurate, acceptable and unacceptable treatment according to the Diagnosis and Treatment Guideline for Primary Care, 2003, and the accuracy rates for SP, GAD, and OCD were 59.3%, 33.3%, and 55.5%, respectively (Figure 2). Out of 37 GPs who diagnosed all the vignettes accurately, only 10 prescribed appropriate treatment.

The percentages of the GPs who preferred to prescribe more than one medication were 19.6%, 35.7%, and 12.9% for SP, GAD, and OCD, respectively. The most commonly seen combinations were SSRI+benzodiazepine (10 GPs), MAO inh+MAO inh (7 GPs) for SP, SSRI+benzodiazepine (21 GPs), SSRI+B bloker (6GPs) for GAD and SSRI+TCA (11 GPs) and SSRI+benzodiazepine (7 GPs) for OCD.

## Discussion and Conclusion

### Summary of main findings

Our vignettes for OCD and SP were diagnosed accurately by 89.0% and 69.4% of the GPs, respectively suggesting that the GPs were aware of these patients; however, they had difficulty in the diagnosis of GAD (22.4%). Although we portrayed our vignettes according to the DSM-IV diagnostic criteria, there can be a point that might have led the GPs to misdiagnosis. However, this factor alone cannot adequately explain why the accuracy of the diagnosis for GAD was so low. This may be due to the high co-morbidity such as depression or any other anxiety disorder which could have been misdiagnosed as panic disorder and depression [4,6,18-21]. Our rates were similar to those of other studies [10,15]. The natural history of GAD being chronic, its controversial nosologic validity over the past 20 years, and the relatively recent interest in its independent impact and treatment in primary care may have contributed to its low rate of recognition [8,10,22]. In daily practice, patients also have a great impact on under-recognition and misdiagnosis of anxiety disorders; however, this will not be discussed here as we used vignettes.

The most commonly used treatment options by primary care providers are pharmacotherapy and psychotherapy [7,22]. While psycho-pharmacotherapy alone and psychotherapy alone were chosen by 13.7-22.4% and 3.9-6.7% of the GPs, respectively, their combination was preferred by 47.5-50.6% of them. In a study conducted in 1997 and 1998, psycho-pharmacotherapy alone and psychotherapy alone were chosen in 54.6% and 2.0% of visits to primary care for anxiety disorders, respectively [23]. However, in a recent study, psycho-pharmacotherapy alone, and psychotherapy alone were used by 21.0% and 7.2% of the GPs, respectively [24]. The rates for the combination treatment had a wide range; 2.8% in one study and 24.5% in another [23,24]. In spite of the difficulties of the interpretation due to methodological differences, it can be commented that there is a tendency towards the combination therapy, and pharmacotherapy has much more impact than psychotherapy, which was also supported by our study [10].

SSRIs are typically considered as the first-line pharmacotherapy in treating patients with anxiety disorders both in secondary and primary care [7,19,24]. In primary care, the study results showed that the prescription of psychotropic drugs and their types do not always reflect the context of a specific diagnosis [13]. In our study, for all the vignettes with different percentages, the GPs preferred SSRIs in the first line. This finding supports the literature also. The percentage of adequate treatment by GPs was 20.9% for any anxiety disorder and 14.8% for GAD in Spain [25]. There is a need for improving the treatment adequacy for anxiety disorders.

Stein et al. reported that primary care physicians recommended referral to a psychiatrist by 37.4% in any anxiety disorder, by 37.7% in GAD and by 36.9% in social phobia [18]. However, our rates were lower than these figures. The referral rates with wrong diagnosis were 19.2%, 22.8%, and 64.7% for social phobia, GAD, and OCD, respectively. The GPs' low referral rates suggested that there is a risk of lack of appropriate treatment in primary care for all anxiety cases, but especially for GAD patients. Although the rates of GPs with post-graduate education in psychiatry for accurate diagnosis were not higher, their accurate treatment rates were higher and referral rates were lower. Although we have no idea about the content of the post-graduate education, treatment was known better than the diagnosis. The post-graduate education, being a part of multifaceted interventions, should not only focus on treatment but also on diagnosis.

It is unlikely to improve the recognition and management of anxiety disorders in primary care by a single intervention. It needs multifaceted interventions including professional and organizational focus on the implementation of post-graduate education, supporting collaborative care with the implementation of guidelines, the integration of primary care and mental health care [7,10,14,15,18].

### Strengths and limitations of the study

Data collection by vignettes can be a method reflecting clinical practice. However, this method has its own limitations. Firstly, our vignettes reflect a primary care presentation fulfilling the DSM-IV diagnostic criteria and this was a necessity due to our guideline, in which the diagnosis of anxiety disorders is based on the DSM-IV. Secondly, vignettes, despite the criteria or guideline they are based on, have concerns about their validity in the exploration of professional decision making [26]. However, a limited number of recent studies support that they are suited for comparative analysis and more effective than chart abstracts [27,28].

We have the limitation of risk for non-respondent bias with a completion rate of 59.4%. We can not say that the respondents are representative of the original sample according to sex and age as we do not have these data. However, we think that this can also suggest an apparent lack of knowledge on anxiety disorders, and also that our results may reflect a bias towards better knowledge.

### Implications for clinical practice and future research

Mental health problems, anxiety disorders in particular, are common in primary care. However, although they are as common as depression, they often receive less attention and they remain unrecognized and untreated [6-10,13-15]. The high rates of co-morbidity with psychiatric disorders and physical illnesses result in varying and misleading presentations. The patients do not usually link their health issues to psychological problems. They even resist these diagnoses and show unwillingness to accept care. In daily practice, these factors coupled with limited time for interviews with patients conspire against GPs' accurate diagnosis of anxiety disorders [15-17]. For the quality of care and knowledge assessments in primary care, vignettes can be used more, especially in countries with limited data like Turkey.

It can be said that the results of our study are not surprising, considering the fact that anxiety disorders are not well-known and presented in practice. We believe that there is a need to question the low rates of accurate diagnosis for anxiety disorders. Effective interventions including post-graduate education and updated guidelines on anxiety disorders should be planned and implemented with their assessments by vignettes.

## Competing interests

The authors declare that they have no competing interests.

## Authors' contributions

MK participated in the design of the study and performed the statistical analysis. OC participated in its design and coordination and helped to draft the manuscript. All authors read and approved the final manuscript.

## Pre-publication history

The pre-publication history for this paper can be accessed here:

http://www.biomedcentral.com/1471-2296/11/30/prepub
